# Arterial Blood Gas Analysis and the Outcome of Treatment in Tricyclic Antidepressants Poisoned Patients with Benzodiazepine Coingestion

**DOI:** 10.1155/2015/232401

**Published:** 2015-01-08

**Authors:** Ahmad Yaraghi, Nastaran Eizadi-Mood, Maryam Katani, Shadi Farsaei, Mahrang Hedaiaty, Seyyed Mohammad Mahdy Mirhosseini, Elham Beheshtian, Ali Mohammad Sabzghabaee

**Affiliations:** ^1^Department of Anesthesiology and Critical Care, School of Medicine, Isfahan University of Medical Sciences, Isfahan, Iran; ^2^Isfahan Clinical Toxicology Research Center, Isfahan University of Medical Sciences, Isfahan, Iran; ^3^Department of Clinical Toxicology, Noor and Ali Asghar (PBUH) University Hospital, Isfahan University of Medical Sciences, Isfahan, Iran; ^4^Department of Clinical Pharmacy and Pharmacy Practice, School of Pharmacy and Pharmaceutical Sciences, Isfahan University of Medical Sciences, Isfahan, Iran

## Abstract

*Background*. Poisoning with tricyclic antidepressants (TCAs) is still a major concern for emergency physicians and intensivists. Concomitant ingestion of other psychoactive drugs especially benzodiazepines with TCAs may make this clinical situation more complex. This study aimed to compare the arterial blood gas (ABG) values and the outcome of treatment in patients with coingestion of TCA and benzodiazepine (TCA + BZD) poisoning and TCA poisoning alone.* Methods*. In this cross-sectional study which was carried out in a tertiary care university hospital in Iran, clinical and paraclinical characteristics of one hundred forty TCA only or TCA + BZD poisoned patients (aged 18–40 years) were evaluated. ABG analysis was done on admission in both groups. Outcomes were considered as survival with or without complication (e.g., intubation) and the frequency of TCA poisoning complications.* Results*. Arterial pH was significantly lower in TCA + BZD poisoning group compared with TCA only poisoning group (7.34 ± 0.08 and 7.38 ± 0.08, resp.; *P* = 0.02). However, other complications such as seizure, and the need for the endotracheal intubation were not significantly different. All patients in both groups survived.* Conclusions*. Concomitant TCA plus BZD poisoning may make the poisoned patients prone to a lower arterial pH level on hospital admission which may potentially increases the risk of cardiovascular complications in TCA poisoning.

## 1. Introduction

Tricyclic antidepressant (TCA) overdose necessitating intensive care remains a major problem and an important critical cause of death due to poisoning around the world [1, 2]. Although new generation of antidepressants is introduced to the market with higher safety profile, physicians in many parts of the world including the Middle East still prescribe older antidepressant drugs including TCAs frequently. These agents have a narrow therapeutic index and regarding the mental condition of patients who take them, an increased prevalence of intentional and accidental overdoses is anticipated [3]. Unfortunately, these drugs alone or in concomitant with other agents including benzodiazepines (BZDs) have been abused for suicidal attempts [4].

Major complications of TCA poisoning involve cardiovascular and central nervous system (CNS) [5, 6]. The main theoretical mechanism of TCAs' cardiovascular toxicity is the inhibition of rapid sodium channels and an effective and practically used treatment for this condition is the administration of sodium bicarbonate [7]. With the rising of TCA poisoning, coingestion of BZDs with TCAs also became more prevalent in recent years, and in practice BZDs were reported to be the most common drugs accompanying cases of TCA overdose in Iran [8]. Coingestion of drugs could lead to different effects on severity and type of the complications, as well as the drug toxicokinetics and metabolism [9, 10]. In a previous study on overdosed patients coingested TCA plus BZD, it was observed that the cardiovascular complications and seizure were seen with less frequency in TCA-alone poisoned cases [10]. This study aimed to compare the arterial blood gas (ABG) values and hemodynamic parameters in patients with coingestion of TCA and benzodiazepine (TCA + BZD) poisoning and TCA poisoning alone. Moreover, patients' outcomes and complications in both groups were also evaluated and compared. To the best of our knowledge, there is not any similar report in the available medical literature.

## 2. Materials and Methods

This cross-sectional study was conducted in Noor and Ali Asghar (PBUH) University Hospital affiliated with Isfahan University of Medical Sciences during 2012-2013. This center which is the major referral medical center for poisoning emergencies for the central part of Iran is facilitated, staffed, and designed for the management of poisoned patients and approximately 400 poisoned patients are admitted to it monthly.

The target population for this study was defined as all symptomatic patients with intentional poisoning in the age range of 18–40 years, having solely TCA poisoning or concomitant coingestion of TCA plus BZD poisoning (TCA + BZD) and without a history of using any other drug in the past two weeks. Patients with a history or underlying disease of cardiovascular, kidney, liver, and chronic obstructive pulmonary diseases and those who had been receiving sodium bicarbonate in another health care center prior to the admission to our center were excluded. Discharge and death of patients before study were also considered as exclusion criteria.

The study protocol was approved by the institutional board of human studies at Isfahan University of Medical Sciences. In addition, after the study was accurately explained to each patient, informed consent was taken from them before their recruitment to this study. If the patient was not able or did not have the capacity for decision making, informed consent for inclusion to this study was taken from the patients' first degree family.

The sample size was estimated for a study power of 80% and a type I error of 5% and also by considering the mean clinically estimated effect size of TCA on ECG and ABG indices. In this regard, the sample size for each group was determined as 70 (total needed number of patients = 140).

On admission of each patient, a careful history was taken and physical examination was performed by a medical toxicologist. Moreover, ABG analysis and ECG were performed at the same time.

All patients underwent continuous heart and O_2_ saturation monitoring during the time period of hospitalization. Any new complication which was developed during monitoring was recorded. Evaluation of cardiovascular symptoms was carried out according to the physical examination findings including vital signs (blood pressure, heart rate, and respiratory rate) and ECG. Moreover, central nervous system manifestations were evaluated according to neurological examinations and the consciousness level. Finally, outcomes were considered as the percent of survival with or without complication (intubation and mechanical ventilation) and cardiovascular or CNS problems regarding TCA poisoning.

For descriptive analysis of the data, continuous variables were expressed as mean ± SD and categorical data as percentage. Kolmogorov-Smirnov test was carried out to assess the normal distribution of continuous variables. Independent Student's *t*-test, chi-square test, and Fisher's exact test were used for statistical analysis of the results. Data processing was performed by SPSS statistical software (SPSS Inc., Chicago, IL, USA) version 20 and a two-tailed *P* value less than 0.05 was considered to establish significance for all statistical tests.

## 3. Results

One hundred forty patients with intentional poisoning by oral ingestion of tablets were studied completely in two groups: TCA poisoning and concomitant ingestion of TCA plus BZD. Each group consisted of 70 patients and the estimated time that elapsed from their poison ingestion was not more than 6 hours. The distribution of gender was different between the two groups (Table 1). Whereas most of the patients were females in TCA group (58.6%), the number of males was higher in the TCA + BZD group (*P* = 0.04). The patients' mean age was not significantly different between groups (26.04 ± 7.6 and 28.97 ± 11.1 years in TCA and TCA + BZD groups, resp.; *P* = 0.07). Amitriptyline and nortriptyline were the most frequently abused drugs in both groups and diazepam and alprazolam were also the most frequently abused benzodiazepines in TCA + BZD group.

Levels of consciousness (awareness, confusion, stupor, and coma) were not significantly different in both groups at the time of admission (*P* = 0.3). Some patients experienced aspiration pneumonia but the groups were not significantly different in this regard (*P* = 0.17) (Table 2).

Comparing the cardiovascular indices obtained from the ECG on admission, the width of QRS complex was not significantly different in the two groups (*P* = 0.67). Statistical significance of the difference between the QT intervals of the patients in two groups was at the borderline (*P* = 0.05).

In addition, according to the pH value in the ABG analysis on admission, the mean pH level in patients with TCA poisoning was significantly higher than that in the patients with concomitant TCA plus BZD poisoning (*P* = 0.02) (Table 3). However, the need for endotracheal intubation was not significantly different in this respect (*P* = 0.8) and about 25% of patients in each group needed intubation. All patients in both groups were survived.

## 4. Discussion

Severe TCA poisoning is an important cause of death among the patients in poisoning emergency rooms around the world and also in our tertiary care poisoning medical center in Iran [11, 12]. Some factors that may underscore the reason of high mortality rate in TCA poisoning are easy availability of the drug for the depressed patients who are susceptible for suicide attempt and the severity of cardiovascular and CNS complications caused by these medications [13].

In the current study, arterial blood gas (ABG) parameters and TCA complications in patients with concomitant TCA and benzodiazepine (TCA + BZD) poisoning and TCA poisoning alone were measured and compared.

Liver cytochrome p450 iso-enzymes are responsible for TCA metabolism and its activity is age dependent. Therefore, patients' ages may have some effects on TCAs' levels and subsequently the manifestations of complications after poisoning [14]. However, in our study patients were not significantly different in terms of the average age (between groups) and age may not be a possible source of bias.

Our results showed that the decrease in consciousness level and seizures were not significantly different in the two groups. These results are somewhat different from similar recently published study which declared that seizure may be less in TCA plus BZD poisoned patients compared with patients intoxicated with TCA alone. BZDs have been used for controlling seizure caused by TCAs. Therefore, lower rate of seizure in previous study may be due to the higher amounts of BZD ingested and lower doses of ingested TCA [10]. It should be mentioned that the amounts of coingested BZDs were not reported in our previous study and also are not available for the current study to compare with each other. So, another clinical study is needed to evaluate the effect of BZDs' doses on the rate of TCAs induced seizure.

Moreover, because acidosis was somewhat compensated and the levels of consciousness were not significantly different in both groups, it was expected that the percent of patients who needed mechanical ventilation was nearly similar in both groups. Our results also show that the amount of coingested BZDs may not be sufficient enough to decrease the level of consciousness in patients with TCA poisoning which could justify the similar rate of seizure between both groups discussed above.

In the current study, vital signs and ECG indices in both groups were not significantly different on admission except for QT interval which is reported at the border line of statistical significance (*P* = 0.05). The mechanism of cardiovascular toxicity caused by TCAs may be attributed to the rapid Na^+^ channel blockade and slowing phase 0 of depolarization and impulse conduction in bundle of Hiss, the Purkinje fibers, and ventricular myocardium [15]. Moreover, rapid Na^+^ channel blockade in heart is sensitive to arterial blood pH, and in this condition acidosis aggravates cardiovascular toxicity in TCA poisoning [10]. In our study according to the reported pH values in the ABG analysis of the patients on admission, the mean pH level in patients with TCA plus BZD poisoning was significantly lower than that in patients with TCA poisoning. This may justify the borderline statistical difference of the prolonged QT interval in patients with TCA plus BZD poisoning. Also, as blood pH value of the patients was increased, the cardiovascular complications and ECG changes appeared to be corrected [16].

Our study had some limitations. First of all, if we could have confirmatory serum levels of TCAs or at least a qualitative screening test in our patients, this could increase the reliability of our results. Secondly, we had samplings from one referral hospital and although this sampling was done in a systematic way it may lead to some concerns for the extrapolation of results. We also relied on the data provided by the patients or close relatives for the past medical history or concomitant drug use. This may have caused recall bias and is not in favor of reliability of our analysis but here in Isfahan we have not a comprehensive national medical recording system to confirm the medical and drug history of patients. Further investigations with confirmatory and screening measurement of TCA serum level and with a more diverse method of multicenter sampling of patients are recommended.

## 5. Conclusion

In conclusion, patients with concomitant TCA plus BZD poisoning may present with a lower pH level on admission and more blood acidity. Since the blood acidity may potentially increase the risk of cardiovascular complications in TCA poisoning, emergency physicians and the intensivists should be aware of these risks and conduct the prompt appropriate intervention for patient management.

## Figures and Tables

**Table 1:** Demographic data of the studied patients in TCA only and TCA plus benzodiazepine ingestion groups.

	TCA (*n* = 70)	TCA + BZD (*n* = 70)	*P* value
Gender			
Male	29	41	0.04^*^
Female	41	29	
Age (years)	26.04 ± 7.6	28.97 ± 11.1	0.07^**^
Frequency and type of the abused TCA			
Amitriptyline	25	31	0.058^*^
Nortriptyline	29	25	
Imipramine	7	8	
Clomipramine	9	6	
Smoking status			
Smokers	17	22	0.34^*^
Nonsmokers	53	48	
Estimated TCA ingested amount (mg)^***^			
<500	21	17	0.40^*^
500–1000	30	38	
>1000	19	15	

^*^Chi-square test; ^**^independent *t*-test; ^***^according to patients' history; TCA: tricyclic antidepressant; BZD: benzodiazepine; results are presented as number of patients or mean ± SD where appropriate.

**Table 2:** Neurological, cardiovascular, and pulmonary characteristics of the studied patients at the time of admission and during hospitalization.

Variables	TCA (*n* = 70)	TCA + BZD (*n* = 70)	*P* value
Delirium			
Present	23 (33)	18 (26)	0.11^*^
Absent	47 (67)	52 (74)	
Level of consciousness			
Alert	11 (15.7)	5 (7.1)	0.30^*^
Lethargic	35 (50)	35 (50)	
Stupor	12 (17.1)	18 (25.7)	
Coma	12 (17.1)	12 (17.1)	
Seizure			
Present	6 (8.6)	8 (10.1)	0.70^*^
Absent	64 (91.4)	62 (89.9)	
Cardiovascular variables			
Systolic blood pressure (mmHg)	107.8 ± 17.4	111.6 ± 21.2	0.45^**^
Diastolic blood pressure (mmHg)	71.4 ± 12.5	72 ± 12.9	0.97^**^
Pulse rate (/min)	91.5 ± 25.9	86.7 ± 22.6	0.13^**^
QRS duration (msec)	0.073 ± 0.02	0.072 ± 0.02	0.67^**^
QT interval (msec)	0.35 ± 0.05	0.37 ± 0.06	0.05^**^
PR interval (msec)	0.19 ± 0.03	0.19 ± 0.04	0.53^**^
Respiratory rate (/min)	18.7 (8.9)	17.9 (4.05)	0.51^**^
Endotracheal intubation			
Performed	17 (24.3)	18 (25.7)	0.08^*^
Not needed	53 (75.7)	52 (74.3)	
Aspiration pneumonia			
Occurred	5 (7.4)	10 (14.8)	0.17^*^
Did not occur	63 (92.6)	58 (85.2)	

^*^Chi-square test; ^**^independent *t*-test; TCA, tricyclic antidepressant; BZD, benzodiazepine; data presented as number of patients (%) or mean ± SD where appropriate.

**Table 3:** Arterial blood gas values of the studied patients at the time of hospital admission.

Variable	TCA (*n* = 70)	TCA + BZD (*n* = 70)	*P* value^*^
pH	7.38 ± 0.08	7.34 ± 0.08	0.02
O_2_ saturation	73.62 ± 30.13	71.00 ± 35.88	0.1
P_a_CO_2_	45.35 ± 6.04	39.00 ± 9.80	0.2
HCO_3_^−^	22.39 ± 4.67	22.03 ± 7.27	0.5
Base excess	4.8 ± 1.26	8.25 ± 0.94	0.1

^*^Independent *t*-test; TCA: tricyclic antidepressant; BZD: benzodiazepine; data presented as mean± SD; P_a_CO_2_: arterial carbon dioxide pressure.
